# Prevalence and abundance of selected genes conferring macrolide resistance genes in COPD patients during maintenance treatment with azithromycin

**DOI:** 10.1186/s13756-020-00783-w

**Published:** 2020-07-28

**Authors:** Remco S. Djamin, Sander Talman, Eefje J. A. Schrauwen, Christian J. H. von Wintersdorff, Petra F. Wolffs, Paul H. M. Savelkoul, Sevim Uzun, René Kerstens, Menno M. van der Eerden, Jan A. J. W. Kluytmans

**Affiliations:** 1 Department t of Respiratory Medicine, Amphia Hospital, Molengracht 21, 4818 CK Breda, The Netherlands; 2 Laboratory for Microbiology and Infection Control, Amphia Hospital, Breda, The Netherlands; 3 Academy for Technology and Environmental Health, Avans University of Applied Sciences, Breda, the Netherlands; 4 Department of Medical Microbiology, Maastricht University Medical Center+, Maastricht, The Netherlands; 5 Orion Statistical Consulting BV, Hilvarenbeek, The Netherlands; 6 Department of Respiratory Medicine, Erasmus Medical Center, Rotterdam, The Netherlands; 7 Julius Center for Health Sciences and Primary Care, University Medical Center Utrecht, Utrecht, The Netherlands

## Abstract

**Objectives:**

Maintenance treatment with macrolide antibiotics has shown to be effective in reducing exacerbations in COPD patients. A major concern with prolonged treatment with antibiotics is the development of bacterial resistance. In this study we determined the effect of azithromycin on the development and acquisition of resistance to macrolides in the nasopharyngeal flora in COPD patients.

**Methods:**

This study was part of the COLUMBUS trial, a randomised, double-blind, placebo-controlled trial to measure the effect of maintenance treatment with azithromycin in 92 COPD patients on the exacerbation rates during a 12-month period. In order to determine resistance to macrolides, we used a targeted metagenomic approach to measure the presence and relative abundance of specific macrolide resistance genes *ermB*, *ermF* and *mefA* in throat samples collected at different time-points during this 12-month period.

**Results:**

There was no increased risk for acquisition of macrolide resistance genes in the azithromycin group compared to the placebo group in COPD patients. However, loss of the macrolide resistance gene *ermB* was increased overtime in the placebo treated group compared to the azithromycin group (*n* = 5 for the placebo group versus *n* = 0 for the azithromycin group at 12 months; *p* = 0.012). The change in relative abundance of the three macrolide-resistance genes showed that all but one (*ermF*) increased during treatment with azithromycin.

**Conclusions:**

The acquisition rate of macrolide resistance genes in COPD patients treated with azithromycin maintenance therapy was limited, but the relative abundance of macrolide resistance genes increased significantly over time compared to placebo.

This study was part of the COLUMBUS trial (Clinicaltrials.gov, NCT00985244).

## Introduction

Chronic obstructive pulmonary disease (COPD) is an important cause of morbidity and mortality [1]. Exacerbations in COPD patients impose a large burden on health care costs and are important events in disease progression [2, 3].

COPD exacerbations are mainly caused by bacterial and viral infections, leading to airway inflammation [4, 5]. Macrolides have antimicrobial, anti-inflammatory and anti-viral effects, which make them potentially useful in reducing COPD exacerbations [6]. Hence, maintenance treatment with macrolide antibiotics has shown to be effective in reducing exacerbations in COPD patients [7–9].

A major concern with prolonged treatment with antibiotics is the development of bacterial resistance [10–12]. Seven major classes of antibiotics have been described, β-lactams and glycopeptides (inhibit cell wall synthesis); macrolides, aminoglyclosides and tetracyclines (protein synthesis); daptomycin (cell membrane function); platensymycin (fatty acid biosynthesis). Bacteria use two mechanisms to achieve resistance: the first is intrinsic resistance and the second is acquired resistance [13]. Intrinsic resistance is the ability to resist the action of specific antibiotics due to inherent structural or functional properties. *Pseudomonas aeruginosa*, for example is resistant for certain classes of antibiotics due to the absence of susceptible target sites for particular antibiotics [14]. Furthermore, *Pseudomonas aeruginosa*, *S. aureus* and e.coli possesses several genes, associated with intrinsic resistance to several classes of antibiotics like B lactams, aminoglyclosides and fluoroqinolones [15, 16].

Bacteria may acquire resistance by antibiotic efflux or poor drug penetration, resulting in reduced concentrations of the intracellular antibiotics. Antibiotics render ineffective due to drug target site modification, due to genetic mutation of the target, posttranslational target modification or antibiotics are inactivated by modification or hydrolysis [17–20].

Gyrase and topoisomerase IV are the two type II topoisomerases utilized in bacteria. Inhibition of those topoisomerases by quinolone based antibiotics prevents uncoiling of DNA strands, thereby preventing replication of bacteria. Acquired resistances against quinolone antibiotics is achieved by mutations in the quinolone binding site of gyrase and topoisomerase IV (gyrA or parC gene) [21], emerging the need of non-quinolone based chemical compounds [12].

The use of macrolides has been associated with the development of macrolide resistance in oral commensal streptococcal microbiota [22]. However, the effect of maintenance treatment with macrolides on resistance in patients with COPD has given controversial results [7–9, 23].

Macrolide resistance can be caused by several mechanisms. Target modification is mediated by one or more rRNA *erm* methylases, which change a site in 23S rRNA [24]. In addition, the *mefA* gene is responsible for a macrolide efflux pump system [25, 26]. Some of these genes are known to persist on mobile genetic elements, which easily facilitate the spread of these resistance genes.

In the present study the randomized control trial by Uzun et al. was futher explored in order to determine the effect of azithromycin maintenance therapy on the dynamics of macrolide resistance genes in the pharyngeal microbiota of COPD patients [9]. We used a targeted (PCR-based) metagenomic approach to determine the presence and relative abundance of specific macrolide resistance genes; *ermB*, *ermF* and *mefA*.

## Methods

### Study design and participants

This study was part of the COLUMBUS trial (Clinicaltrials.gov, NCT00985244), a randomised, double blind, placebo-controlled trial to measure the effect of maintenance treatment with azithromycin in COPD patients on the exacerbation rates during a 12-month period. The study protocol and primary results have been published earlier [9, 17]. Adult patients (≥18 years) with a diagnosis of COPD who had received treatment for three or more exacerbations in the previous year were randomly assigned to receive 500 mg azithromycin or placebo three times a week for 12 months (total of 92 patients).

### Sample collection

During the treatment period, throat samples (e-swabs) were collected at baseline, 6 months and 12 months, as well as during each exacerbation that required admission to the hospital. E-swabs were stored at − 80 °C until molecular analysis was performed.

### Molecular methods

The extraction of DNA was performed from the collected e-swabs™ (COPAN BV), using the EasyMAG (Biomérieux). Real-time PCR was performed to detect and quantify genes responsible for resistance to macrolides; *ermB*, *ermF* and *mefA*. These three specific genes were chosen since these are the most common mobile antibiotic resistance genes that confer macrolide resistance. Amplification of *ermB* was performed as described earlier [27]. Primers to target *mefA* and the forward primer for *ermF* were adapted from earlier described studies [28, 29]. A reverse primer for *ermF* was designed by performing an nBLAST in GenBank for the *ermF* gene sequence (NG_047826.1) and aligning all resulting sequences with > 75% query coverage (identity: 94–100%) using MAFFT (http://mafft.cbrc.jp/alignment/software/), after which a primer homologous to all sequences was chosen.

The 16S ribosomal DNA was amplified as a reference gene to normalize for the amount of bacterial DNA in the samples, using previously described primers [30]. All targets were amplified by using a MyiQ Single-Color Real-Time PCR Detection System (BioRad, Hercules, CA, USA) in 25-μL reactions containing 12.5 μL iQ SYBR Green Supermix (BioRad), 300 nM of both the respective targets forward and reverse primer and 5-μL template DNA. Primer sequences, amplicon sizes and PCR cycling conditions are displayed in Table 1. For all antibiotic resistance gene targets, specificity of the assay was investigated by melting curve analysis of all samples and amplicon sequencing of 10 random positive samples using the PCR primers and an ABI BigDye Terminator v1.1 Cycle Sequencing Kit. Sequencing data were obtained on an ABI 3730 DNA Analyzer (Applied Biosystems, Foster City, CA, USA). All used PCR assays were specifically designed or evaluated for use in metagenomics analyses. During evaluation of the assay, results were confirmed using sequencing. During current analysis each positive signal was manually inspected to be a specific amplification signal by comparing the melting curve to that of the positive control. Samples with a non-identical melting curve were not considered as positive. Efficiencies of the assays were determined to be 103.1% (16S rDNA), 99.7% (*ermF*) and 105.1% (*mefA*).

**Table 1:** PCR conditions and primer sequences

Primer	Sequence 5′ - 3’	Amplicon size (bp)	Cycling conditions
16SrDNA_F	CCTACGGGNGGCWGCAG	465	1 × 95 °C, 3’
16SrDNA_R	GACTACHVGGGTATCTAATCC		35 × 95 °C, 15″; 55 °C, 20″; 72 °C, 30″
*ermB*_F	AAGGGCATTTAACGACGAAACTG	438	1 × 95 °C 3’
*ermB*_R	ATTTATCTGGAACATCTGTGGTATG		40 × 95 °C 15″, 60 °C 20″, 72 °C 30″
*ermF*_F	CGACACAGCTTTGGTTGAAC	120	1 × 95 °C 3′
*ermF*_R	TTTGACACCACTTTGAAAGGAAA		40 × 95 °C 15″, 58 °C 20″, 72 °C 30″
*mefA*	CCTGCAAATGGCGATTATTT	199	1 × 95 °C 3′
*mefA*	AATAGCAAGCACTGCACCAG		40 × 95 °C 15″, 58 °C 20″, 72 °C 30″

### Statistical methods

The prevalence of macrolide genes between the treatment groups was compared using a χ^2^ test. In addition, acquisition and loss of different resistance genes between different treatment groups were compared using a χ^2^ test.

A comparison of the resistance gene abundances between treatment groups was performed based on the samples of both month 6 and month 12. These comparisons were based on the change from baseline, relative to the amount of 16S DNA present, using real-time PCR. These ratios – or fold changes – were calculated for *ermB*, *ermF* and *mefA* using the ΔΔCT method with a Pfaffl modification to correct for PCR efficiency as described earlier [31]. This method is standard to measure the relative change in mRNA expression levels by using real-time PCR. Here, we measure the relative amount of target DNA present rather than measuring mRNA expression. The 16S rDNA was used as the reference gene. In order to perform paired-analysis, multiple throat samples from one patient have to be available in which the presence of the gene of interest was detected. If the gene of interest was not present, this sample was excluded from the paired sample analysis. Ratio’s log-transformed, in order to create a more homogenous population, were compared between treatment arms using the Wilcoxon rank sum test. In addition, descriptive statistics (n, mean, median, SD) and graphical presentations were provided for both time points.

Changes from baseline in relative resistance gene abundances (ratio) were evaluated between samples of month 6 (and month 12) and samples of baseline using the same ΔΔCT method with a Pfaffl modification to correct for PCR efficiency as described earlier (1).

## Results

### Study population

The COLUMBUS trial was a single centre study that took place at the Amphia Hospital (Breda, the Netherlands) between May 19, 2010 and June 18, 2013.

The placebo group consisted of 47 patients and the azithromycin group of 45 patients. The baseline characteristics of these 92 patients are described in Table 2.

**Table 2:** Baseline characteristics

	Azithromycin group (***n*** = 47)	Placebo group (***n*** = 45)
**Male**	22 (46·8%)	18 (40%)
**Age (years)**	64·7 (10·2)	64·9 (10·2)
**Current smoker**	20 (43%)	9 (20%)
**AECOPD in past year**	4·0 (1·2)	4·0 (1·1)
**Hospitalisation due to AECOPD**	1·0 (1·1)	0·7 (0·8)
**Spirometry after bronchodilation**		
**FEV**_**1**_**(L)**	1·1 (0·47)	1·1 (0·43)
**FEV**_**1**_**(% of predicted)**	44·2 (19·3)	45·0 (19·5)
**FVC (L)**	2·9 (0·8)	2·7 (0·92)
**FVC (% of predicted)**	92·5 (22·2)	88·9 (20·3)
**FEV**_**1**_**/FVC (%)**	38·0 (11·7)	40·3 (12·4)
**GOLD stages**		
**I**	2 (4·3%)	3 (6·7%)
**II**	14 (29·8%)	12 (26·7%)
**III**	18 (38·3%)	20 (44·4%)
**IV**	13 (27·7%)	10 (22·2%)

Data are in n (%) or mean (SD), unless otherwise stated. AECOPD = acute exacerbations of COPD. *FEV*_*1*_ Forced expiratory volume in 1 s. *FVC* Forced vital capacity. *GOLD* Global Initiative for Chronic Obstructive Pulmonary Disease

### Prevalence of macrolide resistance genes present in pharyngeal microbiota

At baseline throat samples were taken in 36 (*n* = 36/47; 77%) patients in the placebo group and in 44 (*n* = 44/45; 98%) patients in the azithromycin group. At month 6 and 12 the available samples were, 30 and 27 in placebo group and 34 and 32 in azithromycin group, respectively. The 11 patients in the placebo group and one patient in azithromycin group without a throat sample granted no permission to take an extra throat swab.

Of the 80 patients with a baseline sample taken, 58 (72%) also had a sample at ‘month 6’ and 53 (66%) at ‘month 12’. A total of 43 patients (54%) had samples at both ‘month 6’ and ‘month 12’. This percentage was comparable between treatment arms: 20 (56%) in placebo and 23 (52%) in azithromycin.

The macrolide resistance gene *mefA* was present in all available throat samples at all time points.

Before treatment, prevalence of the macrolide resistance genes *ermF* and *ermB* were respectively 44.4% (*n* = 16/36) and 86.1% (*n* = 31/36) in the placebo group (*n* = 36), and respectively 59.1% (*n* = 26/44) and 97.7% (*n* = 43/44) in the azithromycin group (*n* = 44) (*p* = 0.261 *ermF*, *p* = 0.085 *ermB*) (Table 3).

**Table 3:** Prevalence of *ermF* and *ermB* macrolide resistance genes over time

	*ermF* % (pos/all samples)			*ermB* % (pos/all samples)		
Prevalence	Placebo	Azithromycin	*P* value	Placebo	Azithromycin	*P* value
Baseline	44,4 (16/36)	59,1 (26/44)	0.261	86,1 (31/36)	97,7 (43/44)	0.085
M6	43,3 (13/30)	67,6 (23/34)	0.050	80,0 (24/30)	97,1 (33/34)	0.029*
M12	48,1 (13/27)	68,8 (22/32)	0.109	74,1 (20/27)	100,0 (32/32)	0.002*

*Prevalence of *ermB* is statistically significant in the Azithromycin group at M6 and M12 compared to the Placebo group (Chi-square, Pearson corrected)

After 6 and 12 months of placebo treatment, the *ermF* and *ermB* genes were detected in 43.3% (*n* = 13/30), 80% (*n* = 24/30) at 6 months, and 48.1% (*n* = 13/27) and 74.1% (20/27) at 12 months of the throat samples tested, correspondingly, with no statistical differences regarding the presence of resistance genes between the treatment groups.

Regarding the azithromycin group, the prevalence of the *ermF* and *ermB* genes at 6 months was 67.7% (*n* = 23/34) and 97.1% (*n* = 33/34) versus 68.8% (*n* = 22/32) and 100% (*n* = 32/32) at 12 months (*p* = n.s.). Comparison of the *ermF* prevalence between the placebo and azithromycin groups showed no significant differences at 6 and 12 months (*p* = 0.05 and *p* = 0.109). The difference in prevalence of *ermB* increased significantly over time in the azithromycin group compared to the placebo treated group (*p* = 0.029 6 months, *p* = 0.002; 12 months).

### Loss and acquisition of macrolide-resistance in pharyngeal microbiota during and after treatment with placebo or azithromycin

In the placebo group, 27 patients had throat swabs available from visits at baseline and 6 months while 26-paired samples were available from baseline and 12 months. For the azithromycin group, there were 34 paired samples (from baseline and 6 months) and 30 pairs (from baseline and 12 months).

The loss and acquisition of macrolide resistance genes (*mefA, ermF* and *ermB*) in pharyngeal microbiota before and after treatment of the paired samples is shown in Table 3. During the trial, no differences were detected in the presence of the *mefA* gene in the pharyngeal microbiota.

For the patients without the macrolide genes *ermF* and *ermB* present in their pharyngeal microbiota at baseline (n_*ermF*_ = 15 and n_*ermB*_ = 4 in placebo, n_*ermF*_ = 16 and n_*ermB*_ = 1 in azithromycin)*,* no statistical differences were observed in the acquisition rates between the placebo and azithromycin treated groups.

However, from the patients with the macrolide genes *ermF* and *ermB* present (n_*ermF*_ = 12 and n_*ermB*_ = 23in placebo, n_*ermF*_ = 18 and n_*ermB*_ = 33 in azithromycin) none of the patients treated with azithromycin lost the *ermF* and *ermB* gene over time, while for the placebo group, 1 and 3 patients lost the *ermF* and *ermB* gene after 6 months, respectively. Moreover, in 5 patients in the placebo group, the *ermB* gene was lost after 12 months, therefore, the number of patients that lost the gene was statistically significant higher in the placebo group compared to the azithromycin group (*p* = 0.012).

### Relative gene abundances of the macrolide resistant genes during and after treatment with placebo or azithromycin

**Fig. 1:**
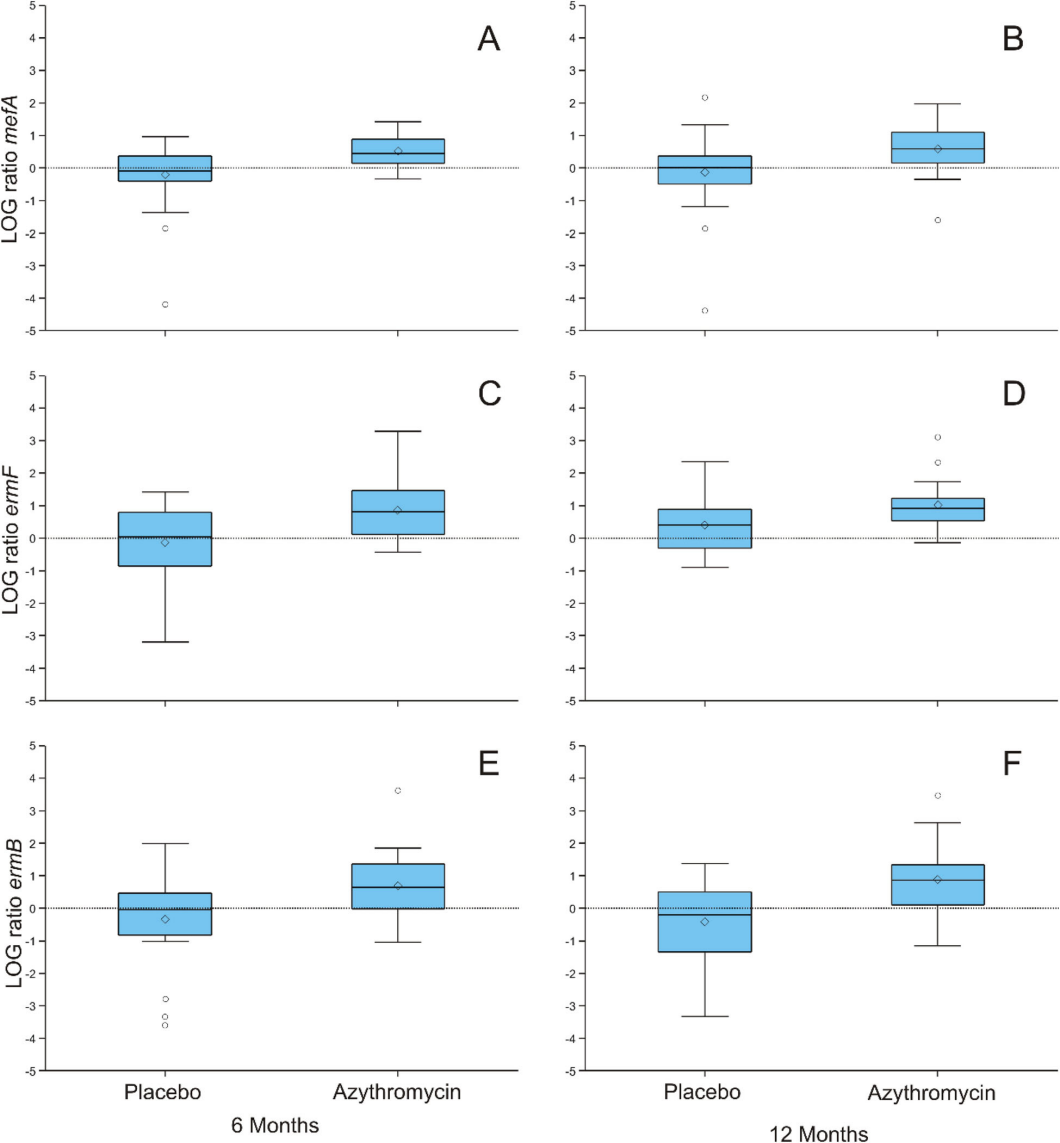
Relative changes in gene abundance at 6 and 12 months after treatment with placebo or azithromycin. Changes related to baseline gene abundance of *mefA (***a**, **b**), *ermF* (**c**, **d**) and *ermB* (**e**, **f**) at 6 and 12 months are shown. Results are visualized in box-plots with median and 10th and 90th percentiles (dots show outliers). The dotted line shows the zero line. Mean logs ratio and statistics are shown in Table 4

A large part of the patients in both groups already had detectable levels of macrolide genes at baseline. This enabled us to compare the relative abundance of the genes in throat samples to determine the effect of the treatment on the abundance of these genes. Figure 1 depicts the overall abundance change of a resistance gene (log-transformed).

The relative gene abundance of *mefA* after 6 months of treatment was substantially higher in the azithromycin versus placebo group − 0.39, *n* = 26; *p* = 0.002) (Fig. 1, Table 4). Determining the overall increase or decrease of the abundance of the *ermF* gene showed that this gene increased over time after treatment with azithromycin (0.86, *n* = 18 M6 and 1.04, *n* = 17 M12) compared to the placebo group (− 0.14, *n* = 11 M6 and 0.15, *n* = 12 M12) as well, which was only significant at 12 months *p* = 0.0124 (Fig. 1, Table 4). With regard to the macrolide gene *ermB,* the relative gene abundance was significantly increased over time in the azithromycin group (0.69, *n* = 33 M6 and 0.89, *n* = 30 M12) compared to the placebo group (− 0.32, *n* = 20 M6 and − 0.42, *n* = 18 M12) after 6 and 12 months of treatment (*p* = 0.01 and *p* = 0.001, respectively) (Fig. 1, Table 4).

**Table 4:** Comparison of mean logs ratios of the different macrolide–resistant genes before, during and after treatment

		Placebo		Azithromycin		
		Mean*	SD	Mean	SD	*P* value**
*mefA*	M6	−0.22 (*n* = 27)	1.00	0.51 (*n* = 34)	0.47	0.0001
	M12	−0.39 *(n* = 26)	1.21	0.33 (*n* = 30)	0.68	0.002
*ermF*	M6	−0.14 (*n* = 11)	1.35	0.86 (*n* = 18)	0.99	0.0687
	M12	0.15 (*n* = 12)	0.94	1.04 (*n* = 17)	0.80	0.0124
*ermB*	M6	−0.32 (*n* = 20)	1.48	0.69 (*n* = 33)	0.93	0.0116
	M12	−0.42 (*n* = 18)	1.33	0.89 (*n* = 30)	1.05	0.0013

^*^mean of the log gene abundance ratio compared to baseline

**p values for comparison of mean abundance at either 6 or 12 months and baseline, by Wilcoxon ranked sum test

## Discussion

During the study, only for the *ermB* gene, a significant difference in prevalence between the azithromycin group and the placebo group was measured over time which was attributed to a loss of this resistance gene within the placebo group. For the *ermF* and *mefA* gene, no differences were detected in the acquisition rates. However, the high prevalence of all resistance genes at baseline, with *mefA* being present in 100% of cases should be taken into consideration. Looking at the relative abundance of the macrolide-resistance genes over-time, a statistical increase of all tested genes in the azithromycin group compared to the placebo group was observed.

Long-term treatment with macrolides might influence the microbiological profile and antibiotic resistance in airways. The acquisition of respiratory pathogens and macrolide resistant microorganisms as a result of maintenance treatment with macrolides in COPD patients has been addressed in three studies [7–9]. It is important to note that these studies did not have the ability to measure quantitative differences over-time. Seemungal and colleagues found no difference in colonization rates with macrolide-resistant organisms between the macrolide and placebo group during1 year of treatment [7]. In contrast with these findings, earlier analysis of our COLUMBUS study found fewer patients in the azithromycin group with macrolide-resistant bacteria in sputum samples compared to those in the placebo group [9]. Albert et al. however, observed an increase in the incidence of colonization with macrolide-resistant organisms in the azithromycin group compared to the placebo group [8]. In summary, it can be stated that there is conflicting evidence about the influence of maintenance treatment with macrolides on the acquisition of macrolide resistant respiratory pathogens in COPD patients. In the current study, only a small difference in acquisition rate of macrolide resistance genes between patients treated with azithromycin or placebo could be demonstrated, nevertheless, a statistical increase in the relative abundance of the tested genes was found. This latter finding suggests that maintenance therapy with azithromycin does influence the presence of macrolide resistance genes, which indicates towards changes in microbiological profile.

To our knowledge, this is the first randomised controlled double blind study in a COPD population, in which the effect of long-term treatment with macrolides on the acquisition and relative abundance of macrolide resistance genes using a targeted metagenomic approach has been evaluated. However, this study has some limitations. Unfortunately, throat samples were not obtained from all patients at regular visits. Furthermore, throat samples were not cultured in order to assess the changes in the microbiological profile and resistance patterns. One additional option would be to assess the microbiota based on the 16SrDNA amplified in the samples. Finally, in this study we focused on three genes, which are involved in macrolide resistance. It is known that more genes and targeted mutations are involved in this process [32–34].

The consequences of this study for daily practice are unclear. The clinical benefit of macrolide maintenance therapy in COPD patients with frequent exacerbations has been demonstrated repeatedly [7–9]. In the most recent update of the GOLD guidelines it is recommended to consider the addition of a macrolide in COPD patients treated with long-acting beta2 agonists/long-acting muscarinic antagonists/inhalation corticosteroids combination, who still have exacerbations [35]. This recommendation is accompanied by the advice that the possibility of developing resistant organisms should be taken into consideration in the decision making.

As indicated, at the start of the study the prevalence of macrolide-resistance genes were already high in throat samples. This may be the result of historical exposure to (macrolide) antibiotics in this specific study population, since only COPD patients with a minimum of three exacerbations in the previous year, have been included in this study. This could be an argument to consider macrolide maintenance treatment only in this specific category of COPD patients. However, this high prevalence has also been observed in a healthy travel population, as shown in the study of von Wintersdorff et al., with an *ermB* gene presence in 99.2% in fecal samples [27].

In conclusion, this study showed that the acquisition rate of macrolide resistance genes in COPD patients treated with azithromycin maintenance therapy was limited, but the relative abundance of macrolide resistance genes increased significantly over time compared to placebo. The clinical implications of these findings are unclear and at this time we consider the observed clinical benefits for this specific group of patients to outweigh the risks of antimicrobial resistance. It is recommended to monitor development of resistance carefully when treating patients for prolonged periods with antibiotics.

## Data Availability

The datasets used and/or analyzed during the study are available from the corresponding author on reasonable request.
